# The climbing flora of India: A comprehensive checklist

**DOI:** 10.12688/f1000research.123818.1

**Published:** 2022-08-25

**Authors:** Vivek Pandi, Kanda Naveen Babu

**Affiliations:** 1 Manipal Centre for Natural Sciences, Centre of Excellence, Manipal Academy of Higher Education (MAHE), Madhava Nagar, Manipal, Udupi, Karnataka, 576104, India; 2 Department of Ecology, French Institute of Pondicherry, Puducherry, Puducherry, 605001, India; 3 Department of Ecology and Environmental Sciences, School of Life Sciences, Pondicherry University, Puducherry, Puducherry, 605014, India

**Keywords:** Lianas, Vines, Diversity, Distribution, Climbing mechanism

## Abstract

The climbing plants in India are listed in detail in this data note. This comprehensive list of climbers was compiled using more than 100 published and unpublished sources that span more than a century. It includes a total of 2,608 species representing 585 genera and 104 spermatophyte plant families. Each species listed in the dataset is categorised according to its degree of woodiness and climbing strategies. The dataset also includes information on The International Union for Conservation of Nature (IUCN) status of all climber species from India. The botanical nomenclature used in the data has been updated to reflect Angiosperm Phylogeny Group (APG) IV classification. Researchers who are conducting ecological, taxonomic, phylogenetic, and evolutionary studies on climbers will be particularly interested in this dataset.

## Introduction

Climbers, among other major growth habits, are likely the most under-collected plant groups. ^1^ Climbers are taxonomically diverse, with representation in 171 plant families, including gymnosperms, pteridophytes, and angiosperms. ^2^ There are also plant families that are entirely made up of climbers, such as Convolvulaceae (55 genera/1,850 species), Cucurbitaceae (97 genera/990 species), and Menispermaceae (72 genera/450 species). Nonetheless, Fabaceae is the most speciose climber family in the paleotropics, ^3^^–^^5^ whereas Apocynaceae and Fabaceae are the most numerous climber families in the Neotropics. ^6^ Climbers are broadly divided into herbaceous (vines) and woody climbers (lianas), which are further subdivided into five major types: twiners, tendril climbers, root climbers, hook climbers, and scramblers, based on their climbing strategies. ^7^

Lianas have long piqued the interest of ecologists due to their unique morphological characteristics, biomechanical properties, anatomical modifications, hydraulic efficiencies and environmental plasticity. ^8^^–^^11^ Climbers frequently lack autonomous vertical growth and must rely on trees for support to reach the canopy. ^12^ Despite this obvious limitation, lianas are extremely important because they structurally bind the forest canopy, maintain forest dynamics, and provide a variety of ecological services. ^1^^,^^13^

Climber research has gained traction over the last two decades, ^14^^–^^16^ but the fundamental question of how many climbers are out there remains unclear at the local and continental scales. One obvious reason for the exclusion of climbers is that climbers have never been treated as distinct plant groups. Climbers were left out of many floristics and ecological inventories due to difficulties in taxonomic assertion, complexity in measurements, and a lack of standard protocols. Furthermore, difficulties in assigning the growth-form and climbing mechanisms among climbers frequently keep them out of the census. Furthermore, the minimum stem size threshold used in ecological inventories essentially excludes vines with thin stems. As a result, we have lower estimates of climber diversity, which may hinder our understanding of their ecological and evolutionary significance. Our current understanding of climber diversity is based on ecological inventories or floristic studies. An integrated approach is, therefore, necessary to precisely estimate the diversity of climbing flora. We came out with a novel approach to estimating the diversity of climbers using Indian flora. India is one of the most biodiverse-rich countries that offers the finest platforms to execute such studies. We developed a methodology for the Indian perspective that can be modified and replicated elsewhere. The baseline data generated by this study will be used in various ecological, taxonomic, phylogenetic, and evolutionary studies on climbers. This would also serve as a precursor to the global climber database.

## Methods

The number of terminologies employed in literature increased in tandem with the interest in climbing plants research. The following terminologies were taken into consideration and used to define climbers in preparing the check-list viz. lianas, climbers, woody climbers, herbaceous climbers, twiners, tendril climbers, root climbers, stragglers, vines, semi-scandent, sub-scandent, climbing sarmentosa shrubs, rambling shrubs, scandent shrub, climbing shrub, rambling climbers, scrambling shrubs, scrambling climbers, robust climbers, hook climbers, and branched climbers. On the other hand, we classified every climber into one of six broad climbing styles: armed-scramblers (SCR-A), unarmed-scramblers (SCR-UA), stem twiners (ST), tendril climbers (TC), root climbers (RC), and hook climbers (HC).

## Data sources, validation, and curation

This data note corresponds to the findings of our study published as Vivek *et al.* ^17^ The current compilation of climbing plants from India is the result of a thorough review of old and recent publications from a variety of sources that were published between 1875 and 2021. We checked 33 published Indian spermatophyte floras for the presence or absence of climbers across the country, including the Andaman and Nicobar Islands. ^17^ Additionally, we consulted a total of 70 research articles focusing on qualitative and quantitative studies of climbers from India and published in peer-reviewed journals. ^17^ Climber information was obtained from a national database that characterised climber diversity in 3,343 micro-plots of 50 m ^2^ each ( BIS (Biodiversity Information System)). Recent records of new species on climbers were also checked against records of new species discoveries published by the Botanical Survey of India between 2008–2020 ( Plant discovers, Botanical Survey of India). Approximately there were 25,000 observations from the initial climber screening (pooled dataset). The most labour-intensive part of building the database was verifying the correctness of the plant species that had been reported by earlier researchers under different names. In such circumstances, we updated the nomenclature to reflect the most recent APG IV classification in order to give proper credit to the valid scientific names. ^18^ We compared the plant names to the World Flora Online (WFO) taxonomic backbone data ( WFO) using the WorldFlora R package ^19^ in accordance with the APG IV classification. ^18^ Ultimately the number of distinct entries produced from the WFO data was considered for preparing the final checklist. The conservation status of every species in the final list was verified as per the revised The International Union for Conservation of Nature (IUCN) criteria and categories. ^20^ We used Microsoft Excel (RRID:SCR_016137), R Project for Statistical Computing (RRID:SCR_001905) Version 4.1.2 ^21^ and ArcGIS for Desktop Basic (RRID:SCR_011081) ver. 10.2. for data analysis. The dataset is available as *Underlying data*. ^22^

## Data availability

### Underlying data

Dryad: Climbing flora of India. https://doi.org/10.5061/dryad.d7wm37q45. ^22^

This project contains the following underlying data:

Climbing flora of India_v1.xlxs (Contains data in a Microsoft Excel spreadsheet of compiled list of climbing plants from India with up-to-date information on nomenclature, family, woodiness, climbing mechanism and IUCN status).

Data are available under the terms of the Creative Commons Zero “No rights reserved” data waiver (CC0 1.0 Public domain dedication).
