# Supplementary material for: The climbing flora of India: A comprehensive checklist
Source: F1000Res. 2022 Aug 25;11:980. [Version 1] doi: 10.12688/f1000research.123818.1 (PMC9579740; doi:10.12688/f1000research.123818.1)
Supplement: Peer review history

# Peer review: Reviewer response for version 1

**DOI:** 10.5256/f1000research.135962.r148672

**Published:** 2022-10-18

**Authors:** Patrick Addo-Fordjour

**Affiliations:** 1 Department of Theoretical and Applied Biology, Faculty of Biosciences, College of Science, Kwame Nkrumah University of Science and Technology, Kumasi, Ghana

This is a fantastic work, which is well written and easy to read and follow. However, there are a few issues that need to be fixed by the authors.

 1. The introduction needs to be supported with more recent articles. The authors stated that Fabaceae is the most species rich family of climbing plants in the paleotropics. This statement is true, but there are more recent and appropriate articles that can be added. For example, the following articles which combine liana data in Africa and Asia, provide more comprehensive and general trends ^1^^,^^2^.

 2. The results of the study must be presented in the paper. The authors should summarise the results of the check list and present them under the title, "Results" This has been done in the abstract, but in the main manuscript, this is conspicuously missing.

 3. Last sentence of the first paragraph of Introduction:

 There are more than 5 climbing modes of climbers. Please see ^1^.

 4. Provide a subtitle for the information under "Methods" section. I suggest: "Climber definition".

Are sufficient details of methods and materials provided to allow replication by others?

Yes

Is the rationale for creating the dataset(s) clearly described?

Yes

Are the datasets clearly presented in a useable and accessible format?

Yes

Are the protocols appropriate and is the work technically sound?

Yes

Reviewer Expertise:

Forest Ecology, Taxonomy

I confirm that I have read this submission and believe that I have an appropriate level of expertise to confirm that it is of an acceptable scientific standard.

## References

[1] : Intercontinental comparison of liana community assemblages in tropical forests of Ghana and Malaysia. *Journal of Plant Ecology* .2016; 10.1093/jpe/rtw082 10.1093/jpe/rtw082

[2] : Liana Assemblages in Tropical Forests of Africa and Southeast Asia: Diversity, Abundance, and Management.5: 10.1007/978-3-319-14592-1_6 81-98 10.1007/978-3-319-14592-1_6

# Peer review: Reviewer response for version 1

**DOI:** 10.5256/f1000research.135962.r150485

**Published:** 2022-09-27

**Authors:** Subbiah Karuppusamy

**Affiliations:** 1 Department of Botany, The Madura College (Autonomous), Madurai, Tamil Nadu, India

1. Major comments:

- What are the data filtering methods used for finalising the data set of climbers?
- Does this study indicate any conservational importance? If so then what basis? 

2. Minor comments: In the introduction section, authors should mentioned the need of this study and why this study is undertaken with justification. Summary must mention the part that is percentage of climbing groups in each Indian state.

 3. General comments: The article is well-written with a quality data set. But the authors failed to classify the various categories of climbers in Indian states with their diversity status. Structure of the article is well planned. The volume of data set is showed the strength of the article.

Are sufficient details of methods and materials provided to allow replication by others?

Yes

Is the rationale for creating the dataset(s) clearly described?

Yes

Are the datasets clearly presented in a useable and accessible format?

Yes

Are the protocols appropriate and is the work technically sound?

Partly

Reviewer Expertise:

Floristics and Biodiversity Conservation

I confirm that I have read this submission and believe that I have an appropriate level of expertise to confirm that it is of an acceptable scientific standard.
